# Direct Measurement of Polarization-Induced Fields in GaN/AlN by Nano-Beam Electron Diffraction

**DOI:** 10.1038/srep28459

**Published:** 2016-06-28

**Authors:** Daniel Carvalho, Knut Müller-Caspary, Marco Schowalter, Tim Grieb, Thorsten Mehrtens, Andreas Rosenauer, Teresa Ben, Rafael García, Andrés Redondo-Cubero, Katharina Lorenz, Bruno Daudin, Francisco M. Morales

**Affiliations:** 1 Department of Materials Science and Metallurgic Engineering, and Inorganic Chemistry, Faculty of Sciences, University of Cádiz, Spain; 2 IMEYMAT: Institute of Research on Electron Microscopy and Materials of the University of Cádiz, Spain; 3 Institut für Festkörperphysik, Universität Bremen, Otto-Hahn-Allee 1, 28359 Bremen, Germany; 4 IPFN, Instituto Superior Técnico, Campus Tecnológico e Nuclear, Universidade de Lisboa, 2695-066 Bobadela LRS, Portugal; 5 Departamento de Física Aplicada y Centro de Micro-Análisis de Materiales, Universidad Autónoma de Madrid, 28049 Madrid, Spain; 6 Univ. Grenoble Alpes, CEA/CNRS Group, “Nanophysique et Semiconducteurs”, F-38000 Grenoble, France

## Abstract

The built-in piezoelectric fields in group III-nitrides can act as road blocks on the way to maximizing the efficiency of opto-electronic devices. In order to overcome this limitation, a proper characterization of these fields is necessary. In this work nano-beam electron diffraction in scanning transmission electron microscopy mode has been used to simultaneously measure the strain state and the induced piezoelectric fields in a GaN/AlN multiple quantum well system.

GaN, AlN, and their related alloys are wide (direct) band gap semiconductors with high thermal and mechanical stability. They have attracted great attention for the fabrication of optoelectronic devices operating in the visible and ultraviolet regions at high power and under harsh environmental conditions^1^,^2^. Multilayered III-N structures are used to change the potential energy along the structure which confines the electrons and holes locally and increases the radiative recombination probability, as the behaviour of excitations in these heterostructures is dominated by strain-induced phenomena at the interfaces^3^.

The quantification of strains at the nanoscale is essential for the development of new electronic devices and for the improvement of existing ones. Besides techniques such as X-ray diffraction and Raman spectroscopy, transmission electron microscopy (TEM) allows strain analyses with a high spatial resolution of a lattice plane distance. Although being a powerful technique to determine the strain state of layered crystals, quantitative analysis of high-resolution transmission electron microscopy (HRTEM) micrographs is hampered due to the structural response of the nanostructure to the thinning of the specimen which usually leads to a bending or buckling of the heterostructure due to differences in the elastic constants of the constituent materials. Moreover, when semiconductor structures are epitaxially grown below their critical layer thickness, the true biaxial strain tends to relax during the specimen thinning (needed for the electron-transparency required in TEM imaging), and is one limitation of HRTEM-based quantifications^4^, among others^5^. Alternatively, dark field electron holography and nano-beam electron diffraction (NBD), which do not require extremely thin specimens (>50 nm) can also be used to measure strains at the nano-level^6^,^7^,^8^. For example, NBD has been used for analysing nano-devices with a strain sensitivity of 0.1%^7^,^8^. One advantage of using NBD over electron holography and HRTEM^9^ is that it can be performed using Scanning-TEM (STEM) probes, offering a practically unlimited field of view.

For bulk group III-nitrides at ambient conditions, the wurtzite (2H) hexagonal phase is energetically more stable for both AlN and GaN and their alloys, compared to the zinc-blende (3C) cubic phase, or to the intermediate possible polytypes. This crystalline structure is non-centrosymmetric with a singular polar axis causing the formation of an electric dipole in the unit cell due to the lack of coincidence of the centre of mass of the negative charge in the N tetrahedrons and the positive charge of the Al/Ga atoms^10^. The dipole gives rise to a spontaneous polarization (P^sp^) across atomic monolayers whose strength is strongly dependent on the difference between the internal cell parameter *u* and its ideal value 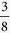 11. Additionally, in the presence of strain, a piezoelectric polarization (P^pz^) is created at the materials interface due to the lattice mismatch that can create fields in the MV/cm range^12^,^13^,^14^. These fields affect the device performance of light emitting diodes and lasers negatively due to the quantum-confined Stark effect^13^,^15^,^16^,^17^. However, in the case of heterostructure field-effect transistors (HFETs) the high sheet carrier concentrations at the interfaces, due to the built in electric fields, is the reason for the outstanding performance of III-N based HFETs^18^. A detailed understanding of the fields is thus essential for the proper design of devices.

Electron holography has been successfully used to study the effects of polarization in group III-nitrides in the recent past^19^,^20^,^21^. However, there it needs a vacuum region near the region of interest which in addition should have a uniform thickness of more than 160 nm in order to achieve reliable data^22^. Thus, the material preparation for these analyses is very demanding and a non-optimal specimen pre-thinning can introduce false strain and bend contours. In-line holography circumvallates most of the limitations inherent in off-axis holography. However, in-line holography requires the use of various defocused images taken at large defocus ranges or a model based approach to relate the complex electron wavefunction to the image intensity by a computational algorithm^23^. And thus, cannot be considered a direct technique. Another alternative for mapping electric fields is NBD, as recent reports have shown that it can be used to map electric fields at atomic scales in extremely thin (<5 nm) specimens^24^,^25^. Müller *et al*.^24^ related diffracted intensities to the expectation value of the momentum transfer caused by the change of the Coloumb potential due to the radial variation of the charge density around the atomic column. Shibata *et al*.^25^, on the other hand used differential phase contrast electron microscopy (DPC) to measure the field induced shift in the transmitted beam using a segmented four-quadrant detector. This is an established method for measuring magnetic fields^26^ and has recently been proposed as a tool to image electric fields^27^. However, quantification of fields by DPC remains a major challenge due to artefacts arising from dynamical diffraction effects.

In this article, we show that NBD combined with an energy filter and STEM conditions can directly and quantitatively profile electric fields in III-N nano-hetrostructures. The main advantages of the proposed method of mapping electric fields are (i) the simple specimen preparation conditions, although uniform thickness in the electron-transparent region of interest is required, and (ii) the simple interpretation of the data which is not affected by influences such as small defocus variations, loss of the high resolution pattern, and exact zone axis orientation. And, contrary to conventional DPC, we are able to record both the primary beam and diffracted beam simultaneously and to determine their positions accurately. For medium variations of electric fields, such as those considered in this work, the primary-beam position is used to determine the direction and magnitude of the electric-field, and the change of the diffracted beam position is used to determine the strain simultaneously. In this way, we can separate the spontaneous and piezoelectric contributions from the total electric field as the piezoelectric constants and composition are known. We verify our results by modelling these fields using the chemical composition profiles measured by high-resolution energy-dispersive X-ray spectroscopy (EDX).

## Results and Discussions

**Figure 1:**
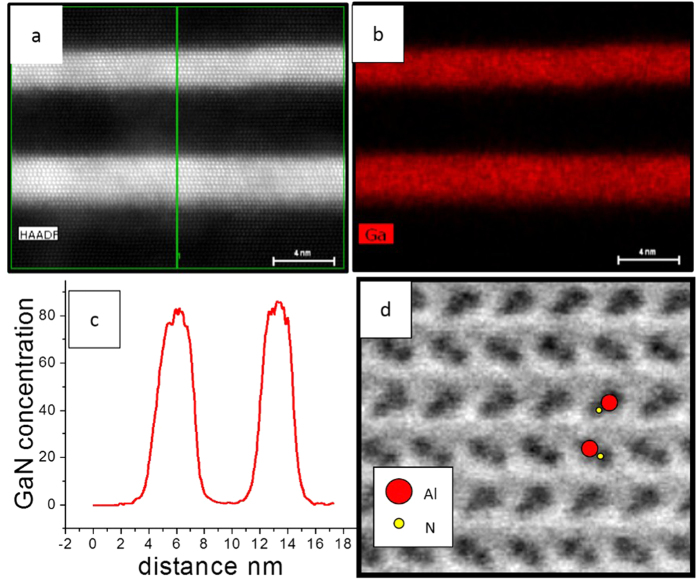
(**a**) HAADF image showing the ROI where the EDX was performed. (**b**) Is the EDX map of the Ga concentration, (**c**) is the concentration profile of Ga obtained by integrating the EDX map perpendicular to the growth direction. (**d**) shows an HR-BF STEM micrograph which was used to determine the [0001] growth direction of the heterostructure.

Figure 1a shows an HAADF image of the region which was used to collect an EDX map (Fig. 1b shows the Ga signal). Figure 1c shows the profile derived by integrating the Ga map perpendicular to the growth direction. From these analyses, the average composition of the ~2 nm multi-quantum wells (MQWs) was determined to be Al_0.2_Ga_0.8_N having sinusoidal-like shapes of graded compositions with a maximum molar fraction of 20% AlN at their centres, and the ~5 nm spacers were found to be pure AlN. This information was used to simulate the strain in the TEM prepared specimen by finite elemental method (FEM) in order to check the effect of stress relaxation on the QWs^28^. The results of FEM modelling showed that for lamella thicknesses over 50 nm, this relaxation is negligible. Since the local thickness of the region where our NBD patterns were recorded is around 65 nm, strain relaxation during sample preparation should not have any appreciable effect on the measured strain. Additionally, high-resolution bright field STEM images (as shown in Fig. 1d) revealed by associating the contrasts to the positions of III-group and N atomic columns that the structure is wurtzite, and grown in the [0001] polar direction with all the III-N material pseudomorphically grown to the relaxed AlN buffer placed beneath.

To detect the deviation of the disc positions in the NBD patterns promoted by the strain at the interfaces, the *Strain Analysis by Nano-Beam Electron Diffraction* (SANBED)^8^,^29^ method was used. Supplementary material S1 explains, in detail, the process to measure the disc positions in NBD patterns. The specimen was tilted, approximately 12 degrees from the [01
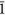
0] zone axis, to excite only the systematic row of 000.2n reflections, next the intensity was scaled logarithmically and the positions of the 0002 and 0000 were measured by cross-correlation.

**Figure 2:**
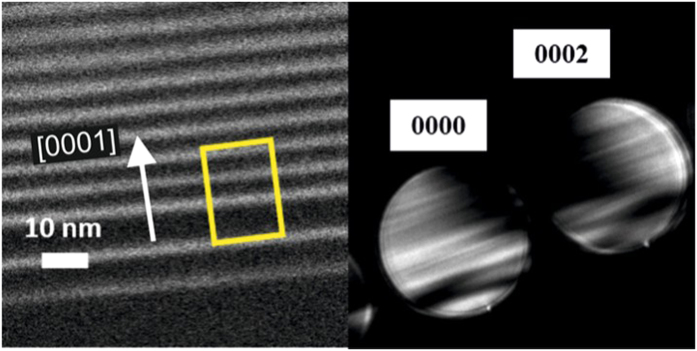
(**a**) HAADF image showing the ROI where the bright regions correspond to AlGaN and the dark regions to AlN where the NBD profile was measured. (**b**) Shows the 0000 and 0002 diffraction discs of a typical pattern collected from the region.

Figure 2a shows the first two quantum wells, which were grown directly after the growth of two test wells, where the NBD patterns were collected. Figure 2b shows a typical pattern acquired by NBD. For the camera length used, the direct- beam and the 0002 beam occupy almost the entire charge-coupled device (CCD), thus allowing for a precise measurement of the beam shift due to the strain or built-in polarization fields.

**Figure 3:**
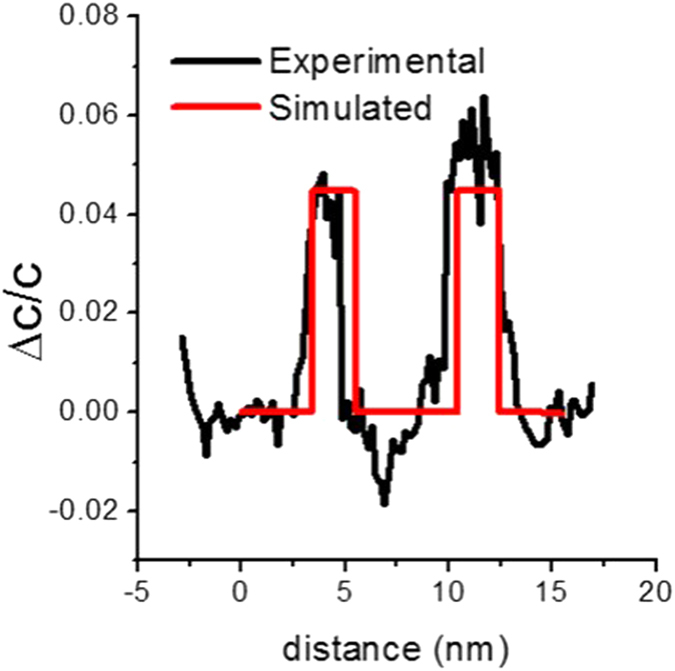
Lattice mismatch profiles acquired by evaluating the shift in the 0002 diffraction discs with respect to the positions of the 0000 discs in the NBD series.

The experimental profiles (calculated by the SANBED method) of the relative lattice parameters with respect to relaxed AlN in the *c* direction (strain *ε*_[0001]_), shown in Fig. 3, are in agreement with simulated data (assuming well-defined 2 nm homogeneous Al_0.2_Ga_0.8_N wells, lattice-matched in the *a*-direction to the 5 nm relaxed AlN spacers, and to the AlN substrate) shown by the red curve.

Assuming this in-plane lattice coherence, the polarization fields can be determined independently from EDX and the NBD data. The piezoelectric polarization P^pz^ is calculated using the following relationship:


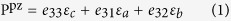


Which simplifies to


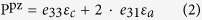


for biaxially strained hexagonal materials, since *ε*_*a*_ = *ε*_*b*_;

Where *e*_33_ and *e*_31_are piezoelectric coefficients^30^, and *ε*_*c*_ and *ε*_*a*_ are the out-of-plane and in-plane strains, respectively. Additionally, *ε*_*c*_ and *ε*_*a*_ are related to each other by the biaxial strain relaxation coefficient:


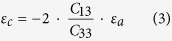


By combining Equations 2 and 3, the piezoelectric polarization takes the form:


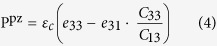


where C_33_ and C_13_ are the relevant elastic constants. The relationship between the strain and composition of a pseudomorphically grown layer, given in Supplementary Material S2, allows us to calculate and compare the piezoelectric polarization independently from composition and strain data.

The values of *c*_0_, *a*_0_, *C*_13_, *C*_33_ along with those of the spontaneous polarization (P^sp^), were calculated by linear interpolation of the AlN and GaN values tabulated in Table 1. Along the line generally accepted for lattice constants, the application of linear interpolations to the elastic constants *C*_ij_ as a function of composition (*x,y,z*) has been found to be very reliable in In_*x*_Al_*y*_Ga_*z*_N materials^31^,^32^,^33^. Supplementary Material S2 details this process.

In S2 the one-to-one relationship between the strain and composition for pseudomorphically grown AlGaN/AlN quantum wells is shown. This allows us to use either strain data to derive the composition or to use composition data to derive the strain, which can then be employed to calculate P^pz^ via equation (4). The piezoelectric constants 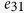, *e*_33_ and the spontaneous polarization P^sp^ were also determined using interpolation of the AlN and GaN values (Table 1 lists the values used for the calculation) in the following way:


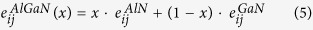



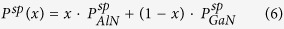


**Figure 4:**
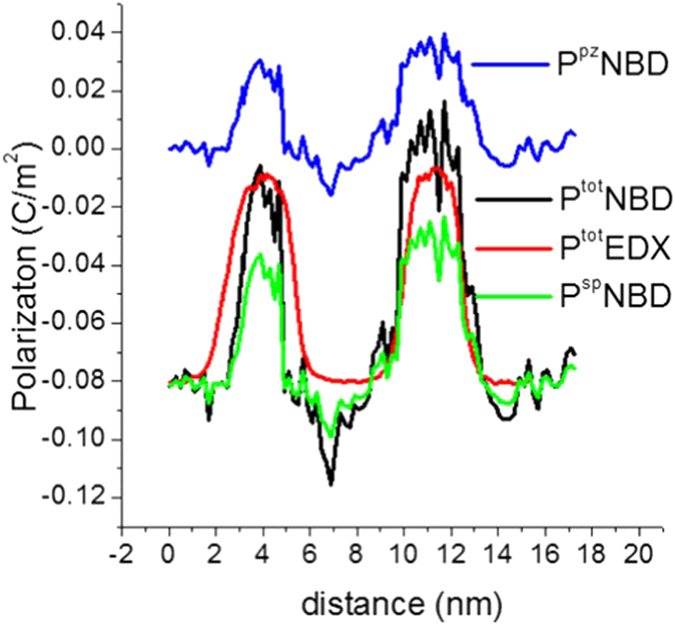
Individual values of the piezoelectric (*P*^*pz*^) and spontaneous (*P*^*ps*^) polarization obtained from NBD, along with the total polarization obtained from both NBD (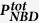) and EDX (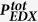).

The negative sign of P^sp^ in Table 1 indicates the direction of the polarization component. For an Al-face structure, the spontaneous polarization vector points towards the [000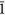] direction (the substrate), therefore a negative value^18^. Similarly, for a tensile strained layer the piezoelectric polarization also points along [000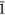]. Since the value of composition 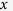
and the strain *ε*_*c*_ can be derived from both EDX and NBD data independently (S2 presents details of this calcualtion) equations (4) and (6) can be derived independently by both methods. Now, the total polarization (P^tot^) is the sum of the two polarizations (P^pz^ + P^sp^). Figure 4 shows P^pz^ and P^sp^ determined using the NBD measurements along with the total polarization deduced from strain measurements extracted from both NBD and EDX independently. Both measurements give reasonably identical values for the total polarization.

**Figure 5:**
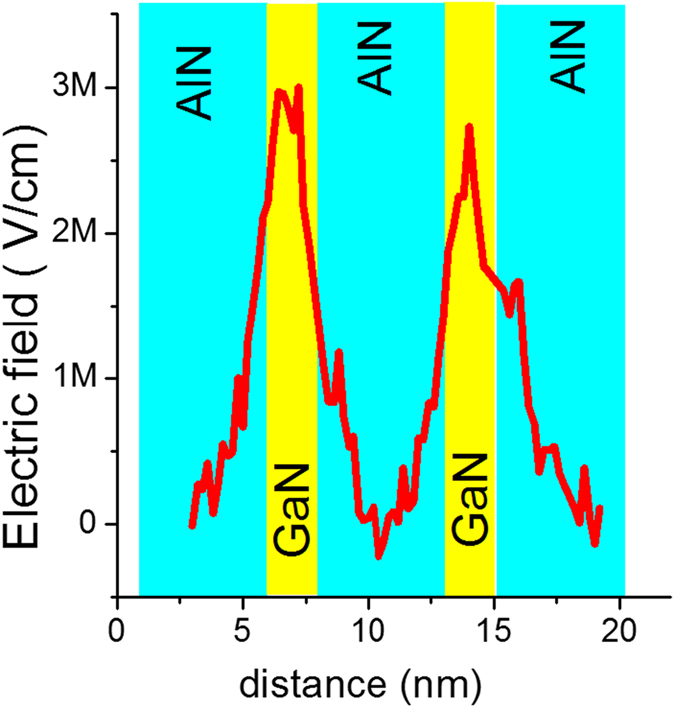
Electric field profile obtained by relating the shift in the 0000 diffraction discs to the electric field in the sample.

To measure the electric field directly, the deflection of the central beam was calculated from its position which has been calibrated by using the 0002 reflection and the known Bragg angle of 7.905 mrad in pure AlN. The field E is then 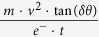 where *m* is the relativistic mass of the electron, *e*^–^ and *v* its charge and velocity respectively, and *t* the thickness of the specimen. Figure 5 shows the electric field calculated from the NBD data set. From here we see that the electric field in the quantum well has a value of around 3 MV/cm, which is 50% less than the value calculated using the strain and composition measured by NBD and EDX, respectively. This drop in the electric field may be ascribed to the charge screening by the background doping concentration, surface barrier potential and to the presence of an electrically ‘dead’ layer that runs around the entire surface of the specimen^34^ which means the total active specimen thickness is lower than the measured 65 nm.

Across interfaces, the polarization induces an accumulation of charge carriers^14^. This induced polarization charge density (*ρ*_*pol*_) can be calculated by numerical differentiation of the polarization profiles, as:


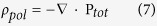


**Figure 6:**
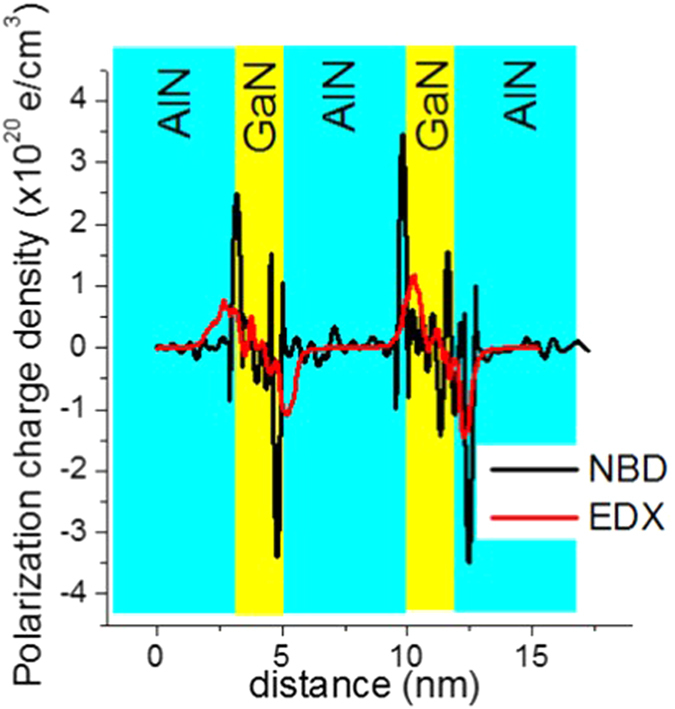
Profiles of polarization induced charge densities obtained from NBD and EDX independently.

The plot of the charge density is shown in Fig. 6. Here we notice that the positive charges accumulate at the upper interface of the quantum wells, while the negative charges accumulate at the bottom interface, from the plot, the maximum polarization charge density is approximately 3 × 10^20 ^e/cm^3^ from the NBD derived data, and close to 1 × 10^20 ^e/cm^3^ derived using the composition profile from EDX measurements. The charge sheet density *σ*_*AlGaN/AlN*_ at the AlGaN/AlN interface is calculated simply by the difference in the total polarization of the barrier and quantum well 
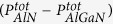
, which gives ~4 × 10^13 ^e/cm^2^. These values are consistent with those reported for an AlN/GaN interface calculated from electron holography data^21^. Fiorentini *et al*.^35^ demonstrated that the magnitude of the total electric field across each well (*E*_*w*_), (in SI units), promoted by the polarization charges can be calculated using the following equation if the screening of these charges is ignored.


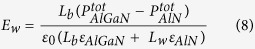


In equation (8) *L*_*b*_ and *L*_*w*_ are the barrier and well heights, respectively and *ε*_0_ is the permittivity of free space, *ε*_*AlGaN*_ and *ε*_*AIN*_ are the relative permittivity of AlGaN and AlN respectively. This gives a field magnitude of 6.44 MV/cm, which is slightly larger than our measurement of 3 MV/cm for the reasons mentioned above.

**Figure 7:**
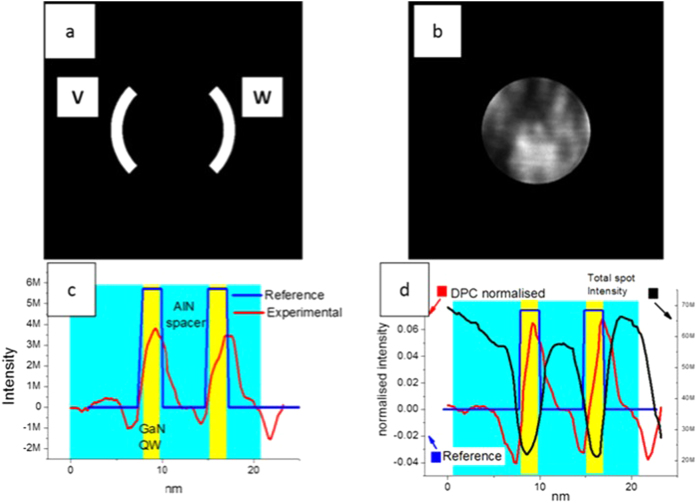
(**a**) shows the image of the virtual detector used for digital-DPC. (**b**) Shows the reference 0000 disc used to calculate the reference signal. (**c**) shows the background subtracted experimental and reference DPC signal. (**d**) Plots the BF (total spot intensity) along with the normalised experimental and reference DPC signal.

Finally, to compare our method to DPC which is an accepted and established method for measuring electric fields, using MATLAB, we created a mask which simulates the function of a two arm segmented detector. This mask can be considered a virtual detector it has a width of 1.55 mrad and an inner angle of 6.85 mrad. The virtual detector subtends an angle of 90° from the centre of 0000-reference disc. Figure 7a shows the image of the virtual detector used for ‘digital-DPC’ measurements, the two arms of the detector are labelled V and W. Figure 7b shows the reference 0000 disc used to calculate the reference signal of the electric-field-induced displacement. Figure 7c shows the signal acquired by both the shift of the reference disc as well as the experimental DPC signal (V-W). As can be noticed from Fig. 7c, the difference in the magnitude of these two measurements is high. This can be attributed to the higher scattering cross-section of Ga as compared to Al, which causes a drop in the intensity of the transmitted beam in the Ga-rich regions. To overcome this, we normalise the DPC intensity by dividing the signal with the total intensity of the 0000 disc or the bright field (BF)-intensity. Figure 7d shows the normalised DPC experimental and reference along with the BF intensity. Taking a closer look at the data we see that neither the shape nor the magnitude of the DPC signal are in agreement with the reference signal. This is due to the complex origin of the features within the transmitted beam which arise from dynamical diffraction contributions. DPC data is thus prone to errors due to the inner structure of diffracted discs^24^. It should be added here that the DPC profiles in Fig. 7d cannot be compared to the electric field profiles acquired by our method, shown in Fig. 5 as the DPC profiles are not quantitative due to the effect of dynamical diffraction in the spots.

## Conclusions

In this work we report the use of NBD to determine the electric field induced by the piezoelectric polarization in a multi-quantum well (MQW) nano-structure. Using NBD both the strain and electric field could be determined independently in one single experiment. Digital-DPC was performed by applying a ‘virtual’ DPC detector to the diffraction patterns, recorded with a CCD camera, in order to compare the two techniques, and it was found that the intensity variations within the transmitted beam strongly contribute to the signal, thus rendering the method ineffective for direct field measurements. Recently in-line holography^36^ was used to measure the piezoelectric charge density at the quantum well interfaces, where most of the limitations imposed by specimen preparation conditions for off-axis holography are not a hindrance. However, as stated earlier, it cannot be considered a direct technique and as in-line holography needs long exposure times (~10 s/image), this can create a problem while measuring the charge density or potential in a specimen due to radiation induced charge migration and a potential build up in the illuminated area of the sample^37^. In contrast, the extraction of strain and electric field by the method discussed here are relatively simple. In the use of NBD this is not a problem as each pattern takes only a second at most and is a technique routinely used in electron microscopy. To conclude, this is the first method to be able to characterize both, the strain as well as electric field from one dataset and has overcome most limitations posed by the other techniques presented above. The experimentally measured electric field at the interface of the quantum well was of the order of 3 MV/cm and the polarization charge density is approximately 3 × 10^20^ e/cm^3^.

## Methods

The MQW sample was grown by molecular beam epitaxy (MBE) on the basal plane of an AlN/sapphire pseudo-substrate. The growth details are reported elsewhere^38^. It was prepared for cross-sectional TEM investigation in a focused ion beam (FIB) facility using a lift-out technique, followed by Ar^+^ etching at 350 V and a 20° oscillation angle with a Technoorg Linda (Model GM IV5) low energy ion milling system to remove the amorphization on the specimen surface due to the FIB etching. The local specimen thickness was determined by comparing the high-angle annular dark-field (HAADF) signal recorded with a detector acceptance angle range of 33–200 mrad (normalized to the intensity of the incoming beam) to the thickness-dependent frozen lattice multi-slice simulations for pure AlN carried out with the STEM simulation program STEMsim^39^. Using this method^40^,^41^, the specimen thickness was determined to be approximately 65 nm. COMSOL was used to carry out FEM analysis.

NBD patterns were collected as described previously^8^, by recording them sequentially along a line profile in STEM mode with the incoming beam parallel to the growth plane in an FEI TITAN 80/300 G1 microscope at Bremen, Germany. Inelastically scattered electrons (plasmons, core excitations) were filtered out using a Tridiem 863 Gatan image filter operated in zero-loss mode with a slit width of 10 eV. Energy-dispersive X-ray (EDX) spectroscopy was performed using the ChemiSTEM Technology on an aberration-corrected FEI Titan 80–300 TEM microscope fitted with a Super-X EDX detector system at the FEI Company, Eindhoven, Netherlands.

## Additional Information

**How to cite this article**: Carvalho, D. *et al*. Direct Measurement of Polarization-Induced Fields in GaN/AlN by Nano-Beam Electron Diffraction. *Sci. Rep.* **6**, 28459; doi: 10.1038/srep28459 (2016).

## Supplementary Material

Supplementary Information

## Figures and Tables

**Table 1:** Constants used in this work, with references.

	*c*_0_ nm	*a*_0_ nm	*e*_33_C/m^2^	*e*_31_ C/m^2^	*C*_13_ GPa	*C*_33_ GPa	*P*^*sp*^ C/m^2^
AlN	0.49809^32^	0.311197^32^	1.46^42^	−0.6^42^	170^32^	404^32^	−0.081^42^
GaN	0.5185^31^	0.31884^31^	0.73^42^	−0.49^42^	101^31^	395^31^	−0.029^42^

## References

[b1] Crawford M. H. LEDs for Solid-State Lighting: Performance Challenges and Recent Advances. IEEE J. Sel. Topics Quantum Electron. 15, 1028–1040, 10.1109/jstqe.2009.2013476 (2009).

[b2] Lien D.-H. . Harsh photovoltaics using InGaN/GaN multiple quantum well schemes. Nano Energy 11, 104–109 (2015).

[b3] Soh C. B. . Influence of composition pulling effect on the two-dimensional electron gas formed at AlyInxGa1-x-yN/GaN interface. J. Appl. Phys. 98, 103704, 10.1063/1.2132090 (2005).

[b4] Wang Z. . Investigation of Strain and Thin Film Relaxation in Ge_x_Si_1−x_/Si Strained-Layer Superlattice by Dark-Field Electron Holography. Mater. Trans., JIM 53, 2019–2022, 10.2320/matertrans.M2012138 (2012).

[b5] Rosenauer A., Gerthsen D. & Potin V. Strain state analysis of InGaN/GaN–sources of error and optimized imaging conditions. Phys. Status Solidi A 203, 176–184, 10.1002/pssa.200563519 (2006).

[b6] Hytch M., Houdellier F., Hue F. & Snoeck E. Nanoscale holographic interferometry for strain measurements in electronic devices. Nature 453, 1086–1089 (2008). 18563161 10.1038/nature07049

[b7] Armigliato A., Frabboni S. & Gazzadi G. C. Electron diffraction with ten nanometer beam size for strain analysis of nanodevices. Appl. Phys. Lett 93, 10.1063/1.3003581 (2008).

[b8] Müller K. . Strain Measurement in Semiconductor Heterostructures by Scanning Transmission Electron Microscopy. Microsc Microanal 18, 995–1009, 10.1017/S1431927612001274 (2012). 23026441

[b9] Hähnel A. . Nano-beam electron diffraction evaluation of strain behaviour in nano-scale patterned strained silicon-on-insulator. Phys. Status Solidi C 8, 1319–1324, 10.1002/pssc.201084007 (2011).

[b10] Bernardini F., Fiorentini V. & Vanderbilt D. Spontaneous polarization and piezoelectric constants of III-V nitrides. Phys. Rev. B 56, R10024–R10027 10.1103/PhysRevB.56.R10024 (1997).

[b11] Lähnemann J. . Direct experimental determination of the spontaneous polarization of GaN. Phys. Rev. B 86, 081302 10.1103/PhysRevB.86.081302 (2012).

[b12] Simon J. . Direct comparison of recombination dynamics in cubic and hexagonal GaN/AlN quantum dots. Phys. Rev. B 68, 035312 10.1103/PhysRevB.68.035312 (2003).

[b13] Yu E. T., Dang X. Z., Asbeck P. M., Lau S. S. & Sullivan G. J. Spontaneous and piezoelectric polarization effects in III-V nitride heterostructures. J. Vac. Sci. Technol. B 17, 1742–1749, 10.1116/1.590818 (1999).

[b14] Ambacher O. . Two-dimensional electron gases induced by spontaneous and piezoelectric polarization charges in N- and Ga-face AlGaN/GaN heterostructures. J. Appl. Phys 85, 3222–3233 10.1063/1.369664 (1999).

[b15] Doan Nhat Q., Nguyen Huyen T. & Nguyen Thanh T. Electron scattering from polarization charges bound on a rough interface of polar heterostructures. J. Appl. Phys 109, 113711, 10.1063/1.3592187 (2011).

[b16] Redondo-Cubero A. . Selective ion-induced intermixing and damage in low-dimensional GaN/AlN quantum structures. Nanotechnology 24, 505717, 10.1088/0957-4484/24/50/505717 (2013). 24285147

[b17] Tetsuya T. . Quantum-Confined Stark Effect due to Piezoelectric Fields in GaInN Strained Quantum Wells. Jpn. J. Appl. Phys 36, L382, 10.1143/JJAP.36.L382 (1997).

[b18] Ambacher O. & Foutz B. Two dimensional electron gases induced by spontaneous and piezoelectric polarization in undoped. J. Appl. Phys 87, 334, 10.1063/1.371866 (2000).

[b19] Wei Q. Y., Wu Z. H., Ponce F. A., Hertkorn J. & Scholz F. Polarization effects in 2-DEG and 2-DHG AlGaN/AlN/GaN multi-heterostructures measured by electron holography. Phys. Status Solidi B 247, 1722–1724, 10.1002/pssb.201046198 (2010).

[b20] Wu Z. H. . Mapping the electrostatic potential across AlGaN∕AlN∕GaN heterostructures using electron holography. Appl. Phys. Lett 90, 10.1063/1.2431716 (2007).

[b21] Zhou L. . Polarization field mapping of Al_0.85_In_0.15_N/AlN/GaN heterostructure. Appl. Phys. Lett 94, 121909, 10.1063/1.3108084 (2009).

[b22] Han M.-G. . Sample Preparation for Precise and Quantitative Electron Holographic Analysis of Semiconductor Devices. Microsc Microanal 12, 295–301, 10.1017/S1431927606060351 (2006). 16842641

[b23] Ozsoy-Keskinbora C., Boothroyd C. B., Dunin-Borkowski R. E., van Aken P. A. & Koch C. T. Hybridization approach to in-line and off-axis (electron) holography for superior resolution and phase sensitivity. Sci. Rep. 4, 10.1038/srep07020 (2014). PMC4228327 25387480

[b24] Müller K. . Atomic electric fields revealed by a quantum mechanical approach to electron picodiffraction. Nat Commun 5, 10.1038/ncomms6653 (2014). PMC4275586 25501385

[b25] Shibata N. . Differential phase-contrast microscopy at atomic resolution. Nat Phys 8, 611–615, 10.1038/nphys2337 (2012).

[b26] Chapman J. N., Batson P. E., Waddell E. M. & Ferrier R. P. The direct determination of magnetic domain wall profiles by differential phase contrast electron microscopy. Ultramicroscopy 3, 203–214, 10.1016/S0304-3991(78)80027-8 (1978). 358526

[b27] Lohr M. . Differential phase contrast 2.0–Opening new “fields” for an established technique. Ultramicroscopy 117, 7–14, 10.1016/j.ultramic.2012.03.020 (2012). 22634135

[b28] Tillmann K. . Finite element analysis of the strain induced vertical ordering of islands and determination of compositional modifications in LPCVD-grown Ge_x_Si_1−x_-Si bilayers on Si(001). Philos. Mag A 80, 255–277, 10.1080/01418610008212052 (2000).

[b29] Müller K. . STEM strain analysis at sub-nanometre scale using millisecond frames from a direct electron read-out CCD camera. JPCS 471, 012024 10.1088/1742-6596/471/1/012024 (2013).

[b30] Ambacher O. . Two dimensional electron gases induced by spontaneous and piezoelectric polarization in undoped and doped AlGaN/GaN heterostructures. J. Appl. Phys 87, 334–344, 10.1063/1.371866 (2000).

[b31] Morales F. M. . Determination of the composition of In__x__Ga_1−x_N from strain measurements. Acta Mater 57, 5681–5692, 10.1016/j.actamat.2009.07.063 (2009).

[b32] Mánuel J. M. . Structural and compositional homogeneity of InAlN epitaxial layers nearly lattice-matched to GaN. Acta Mater 58, 4120–4125, 10.1016/j.actamat.2010.04.001 (2010).

[b33] Morales F. M. . Evaluation of interpolations of InN, AlN and GaN lattice and elastic constants for their ternary and quaternary alloys. J Phys D Appl Phys 46, 245502 10.1088/0022-3727/46/24/245502 (2013).

[b34] Twitchett A. C., Dunin-Borkowski R. E. & Midgley P. A. Quantitative Electron Holography of Biased Semiconductor Devices. Phys. Rev. Lett 88, 238302 10.1103/PhysRevLett.88.238302 (2002). 12059403

[b35] Fiorentini V., Bernardini F., Della Sala F., Di Carlo A. & Lugli P. Effects of macroscopic polarization in III-V nitride multiple quantum wells. Phys. Rev. B 60, 8849–8858 10.1103/PhysRevB.60.8849 (1999).

[b36] Song K. . Correlative High-Resolution Mapping of Strain and Charge Density in a Strained Piezoelectric Multilayer. Adv. Mater. Interf 2, n/a-n/a, 10.1002/admi.201400281 (2015).

[b37] Cazaux J. Correlations between ionization radiation damage and charging effects in transmission electron microscopy. Ultramicroscopy 60, 411–425, 10.1016/0304-3991(95)00077-1 (1995).

[b38] Adelmann C. . Growth and optical properties of GaN/AlN quantum wells. Applied Physics Letters 82, 4154–4156, 10.1063/1.1581386 (2003).

[b39] Rosenauer A. & Schowalter M. STEMSIM–a New Software Tool for Simulation of STEM HAADF Z-Contrast Imaging In Microscopy of Semiconducting Materials 2007 Vol. 120 *Springer Proceedings in Physics* (eds Cullis A. G. & Midgley P. A. ) Ch. 36, 170–172 (Springer, 2008).

[b40] Rosenauer A. . Composition mapping in InGaN by scanning transmission electron microscopy. Ultramicroscopy 111, 1316–1327, 10.1016/j.ultramic.2011.04.009 (2011). 21864772

[b41] Rosenauer A. . Measurement of specimen thickness and composition in using high-angle annular dark field images. Ultramicroscopy 109, 1171–1182, 10.1016/j.ultramic.2009.05.003 (2009). 19497670

[b42] Smart J. A. . AlGaN/GaN heterostructures on insulating AlGaN nucleation layers. Appl. Phys. Lett 75, 388–390 10.1063/1.124384 (1999).

