# Supplementary material for: Direct Measurement of Polarization-Induced Fields in GaN/AlN by Nano-Beam Electron Diffraction
Source: Sci Rep. 2016 Jun 28;6:28459. doi: 10.1038/srep28459 (PMC4923855; doi:10.1038/srep28459)
Supplement: Supplementary Information [file srep28459-s1.pdf]

Supplementary information for:

Direct Measurement of Polarization-Induced Fields in GaN/AlN by Nano-Beam Electron Diffraction

Daniel Carvalho[1,21], Knut Müller-Caspary[3], Marco Schowalter[3], Tim Grieb[3], Thorsten Mehrtens[3] Andreas Rosenauer[3], Teresa Ben[1,2], Rafael García[1,2], Andrés Redondo-Cubero[4,5], Katharina Lorenz[4], B Daudin[6], Francisco M. Morales[1,2],
[1]Department of Materials Science and Metallurgic Engineering, and Inorganic Chemistry, Faculty of Sciences, University of Cádiz, Spain
[2]IMEYMAT: Institute of Research on Electron Microscopy and Materials of the University of Cádiz, Spain
[3]Institut für Festkörperphysik, Universität Bremen, Otto-Hahn-Allee 1, 28359 Bremen, Germany
[4] IPFN, Instituto Superior Técnico, Campus Tecnológico e Nuclear, Universidade de Lisboa, 2695-066 Bobadela LRS, Portugal
[5] Departamento de Física Aplicada y Centro de Micro-Análisis de Materiales, Universidad Autónoma de Madrid, 28049 Madrid, Spain
[6]Univ. Grenoble Alpes, CEA/CNRS Group, “Nanophysique et Semiconducteurs”, F-38000 Grenoble, France

S1: Measurement of spot movement using SANBED:

In this section we present the methodology of the *Strain Analysis by Nano-Beam Electron Diffraction* (SANBED) [1,2] technique used to measure the deflection of the direct 0000 beam as well as the strain in the wells.

The post processing involved in order to measure the deflection of the direct 0000 beam as well as the strain in the wells are listed below:

1) The diffraction pattern was scaled logarithmically. This is done to better separate the background noise from spot intensity. Thus, allowing more accurate measurement to the disc position.[2]
2) Two masks were applied around the spots of interest. The 0000 and 0002 in our case. For the purpose of reducing computational time regions of interest are

[1] author to whom correspondence should be addressed: daniel.carvalho@uca.es

selected and only these regions are considered while processing the image. Figure S1-1 illustrates the selection of the spots (yellow) and regions of interest (white)

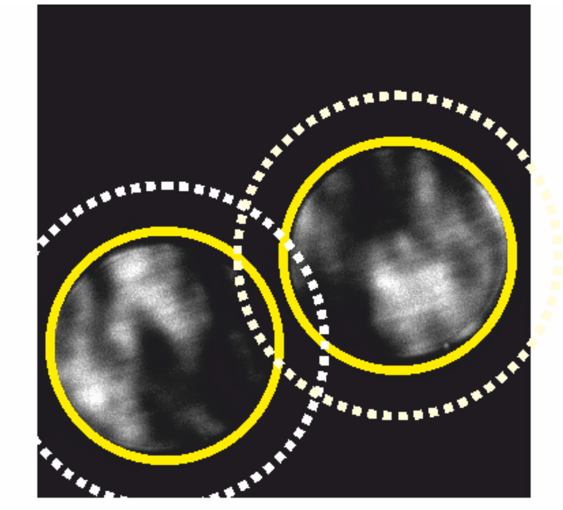

Figure S1-1 shows the selection of the masks in yellow, solid circles and the region of interest outlined by a white dotted line.

3) Cross-correlation was then used to find the position of the diffraction discs in the image series. Figure S1-2 shows a typical cross-correlation map acquired after cross-correlating an entire DP with one of the masks.
4) The pixel with the maximum value was selected and a 10 pixel x 10 pixel region was selected around the pixel. As seen in figure S1-2
5) The intensity of the region was then integrated along the x and y axis and the fitted to a polynomial (not shown here). The maxima of each polynomial is taken as the position of the diffraction spot for that DP.

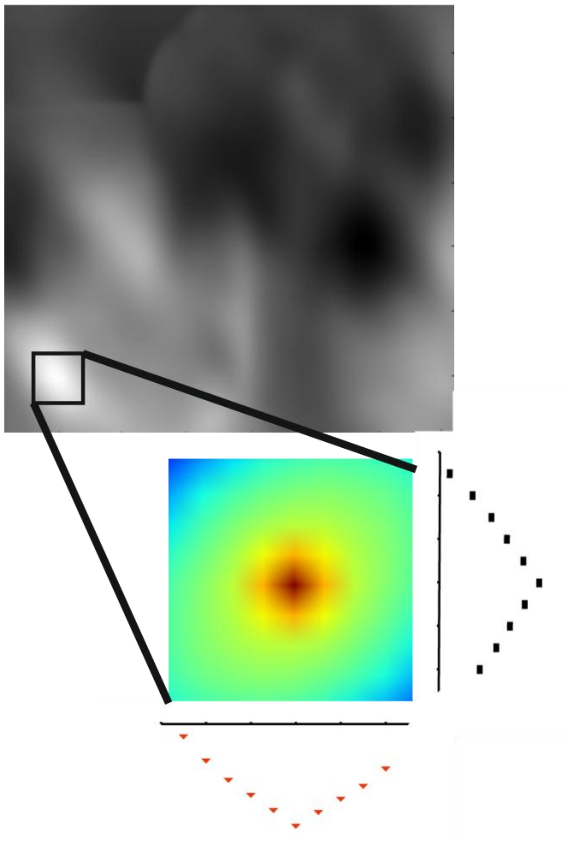

Figure S1-2 shows the cross-correlation map of acquired by using the reference discs as a template. The inset shows a 10 pixel x 10 pixel region around the pixel with highest intensity along with profiles acquired by integrating along the rows and columns of this region.

6) Once the position of the diffraction spot is calculated, the relative change in the c parameter ($\Delta c = c - c_0^{AlN}$) of the III-N unit cell, with respect to that of the relaxed AlN lattice constant c_0^{AlN} was calculated using the expression:

$$\frac{\Delta c}{c_0^{AlN}} = \frac{d^{ref}}{d} - 1 = \varepsilon_{[0001]}$$

Where d^{ref} is the distance between the 0000 and 0002 disc position in the pure AlN reference diffraction pattern, and d is the corresponding distance in each pattern of the series taken along a line with a direction perpendicular to the interfaces.

7) For detecting the deflection of the electron beam due to the electric fields in the sample only the change in position of the transmitted beam (0000) was considered.

1 Müller, K. *et al.* STEM strain analysis at sub-nanometre scale using millisecond frames from a direct electron read-out CCD camera. *Journal of Physics: Conference Series* **471**, 012024, doi:doi:10.1088/1742-6596/471/1/012024 (2013).

2 Müller, K. *et al.* Strain Measurement in Semiconductor Heterostructures by Scanning Transmission Electron Microscopy. *Microscopy and Microanalysis* **18**, 995-1009, doi:doi:10.1017/S1431927612001274 (2012).

S2: Strain-composition and composition-strain relation:

In this section we present the relation between the strain and composition of pseudomorphically grown AlGaN/AlN quantum wells. Because of the one-to-one relation between them, the strain in the well can be calculated from composition data and the composition of the well can be calculated from strain data.

For a cristal if no force is applied in the growth direction and the crystal is free to expand or contract, the strain along the c-axis and basal plane is given by:

$$\varepsilon_c = -2\frac{13}{C_{33}}\varepsilon_a \qquad (1)$$

Where $\varepsilon_a = \frac{a-a_0}{a_0}$ and $\varepsilon_c = \frac{c-c_0}{c_0}$ are the relative changes in the lattice constants a and c with respect to the relaxed crystal a_0 and c_0 . For a pseudomorphicly grown layer the lattice parameter a of the crystal is equal to the lattice parameter of layer below. Thus in the case of an AlGaN grown on an AlN substrate, the AlGaN layer will have a strained a parameter which is equal to a parameter of AlN, in other words;

$$\varepsilon_a = \frac{a^{AlN} - a_0^{AlGaN}}{a_0^{AlGaN}} \qquad (2)$$

Where a^{AlN} is the lattice parameter of the AlN crystal and a_0^{AlGaN} that of a relaxed AlGaN crystal. Substituting equation (2) in (1) we can calculate the strain ε_c in the AlGaN layer. In order to calculate the strain ε_c for the entire $Al_xGa_{(1-x)}N$ composition range, the lattice parameters of the relaxed crystal should be calculated using Vegard's law

$$c_0^{AlGaN}(x) = x \cdot c_0^{AlN} + (1-x) \cdot c_0^{GaN} \qquad (3)$$

$$a_0^{AlGaN}(x) = x \cdot a_0^{AlN} + (1-x) \cdot a_0^{GaN} \qquad (4)$$

The elastic constants C_{13} and C_{33} are calculate by interpolating the values between the the GaN and AlN elastic constants.

$$C_{ij}^{AlGaN}(x) = x \cdot C_{ij}^{AlN} + (1 - x) \cdot C_{ij}^{GaN} \quad (5)$$

Where C_{ij}^{AlGaN} is the elastic constant of the pseudomorphic layer and C_{ij}^{AlN} and C_{ij}^{GaN} are the elastic constants of AlN and GaN crystals respectively.

Using equations (1) to (5) calculation of a strain-composition relationship is straightforward. Figure S2 illustrates this relationship. From the figure S2 one can appreciate the one-to-one linear relationship between the strain and composition for a pseudomorphic AlGaN layer. This relationship allows us to estimate the strain state of the layer for known compositions and the composition of the layer if the c parameter is known.

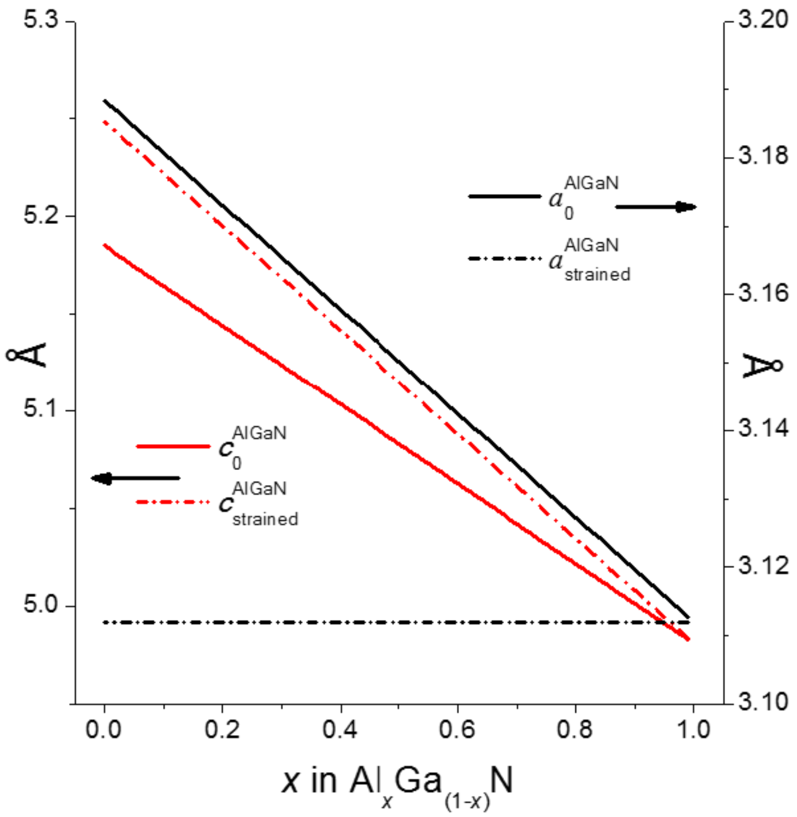

Figure S2 Theoretical values for a and c lattice parameters of AlGAN using Vegard's law for a relaxed bulk crystal and a strained pseudomorphic layer grown over AlN.
